# 3,6-Dichloro-*N*-(4,6-dichloro­pyrimidin-2-yl)picolinamide

**DOI:** 10.1107/S1600536808010325

**Published:** 2008-04-18

**Authors:** Shan-Shan Zhang, Yue Zhuang, Xian-Hong Yin, Kai Zhao, Cui-Wu Lin

**Affiliations:** a College of Chemistry and Ecological Engineering, Guangxi University for Nationalities, Nanning 530006, People’s Republic of China; b College of Chemistry and Chemical Engineering, Guangxi University, Nanning 530006, People’s Republic of China

## Abstract

In the title compound, C_10_H_4_Cl_4_N_4_O, the pyridine and pyrimidine rings are nearly perpendicular to each other, the dihedral angle between them being 86.60 (10)°. In the crystal structure, the N and O atoms in the amide group are involved in inter­molecular hydrogen bonds, forming a one-dimensional chain along the *c* axis.

## Related literature

For related literature, see: Liu *et al.* (2005 ▶); Śladowska *et al.* (1999 ▶).
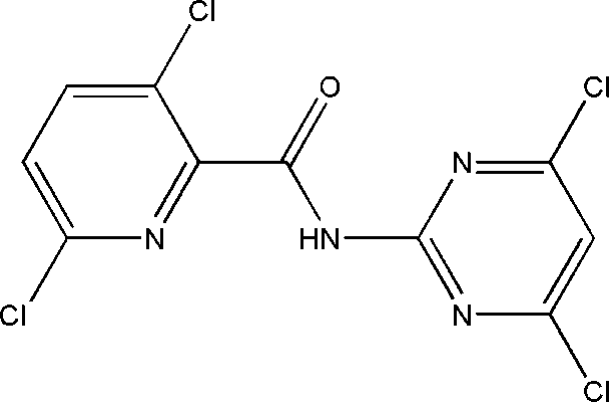


## Experimental

### 

#### Crystal data

C_10_H_4_Cl_4_N_4_O
*M* *_r_* = 337.97
Monoclinic, 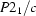

*a* = 10.9313 (13) Å
*b* = 13.3682 (14) Å
*c* = 9.3846 (10) Å
β = 112.576 (1)°
*V* = 1266.3 (2) Å^3^
*Z* = 4
Mo *K*α radiation
μ = 0.93 mm^−1^
*T* = 293 (2) K
0.48 × 0.43 × 0.40 mm

#### Data collection

Bruker SMART 1000 diffractometer
Absorption correction: multi-scan (**SADABS**; Sheldrick, 1996 ▶) *T*_min_ = 0.648, *T*_max_ = 0.690
15165 measured reflections
2494 independent reflections
2132 reflections with *I* > 2σ(*I*)
*R*_int_ = 0.032

#### Refinement

*R*[*F* ^2^ > 2σ(*F* ^2^)] = 0.033
*wR*(*F* ^2^) = 0.096
*S* = 1.02
2494 reflections
172 parameters
H-atom parameters constrained
Δρ_max_ = 0.34 e Å^−3^
Δρ_min_ = −0.30 e Å^−3^

### 

Data collection: *SMART* (Siemens, 1996 ▶); cell refinement: *SAINT* (Siemens, 1996 ▶); data reduction: *SAINT*; program(s) used to solve structure: *SHELXS97* (Sheldrick, 2008 ▶); program(s) used to refine structure: *SHELXL97* (Sheldrick, 2008 ▶); molecular graphics: *SHELXTL* (Sheldrick, 2008 ▶); software used to prepare material for publication: *SHELXTL*.

## Supplementary Material

Crystal structure: contains datablocks I, global. DOI: 10.1107/S1600536808010325/is2279sup1.cif

Structure factors: contains datablocks I. DOI: 10.1107/S1600536808010325/is2279Isup2.hkl

Additional supplementary materials:  crystallographic information; 3D view; checkCIF report

## Figures and Tables

**Table 1:** Hydrogen-bond geometry (Å, °)

*D*—H⋯*A*	*D*—H	H⋯*A*	*D*⋯*A*	*D*—H⋯*A*
N1—H1⋯O1^i^	0.86	2.09	2.937 (2)	170

Symmetry code: (i) 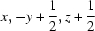.

**Fig. 1.**
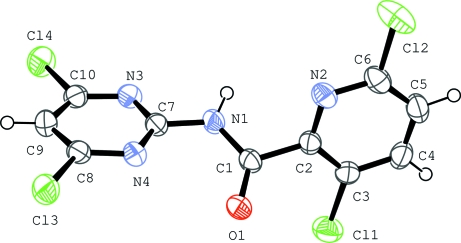
The molecular structure of the title compound, showing 50% probability displacement ellipsoids and the atom-numbering scheme.

**Fig. 2.**
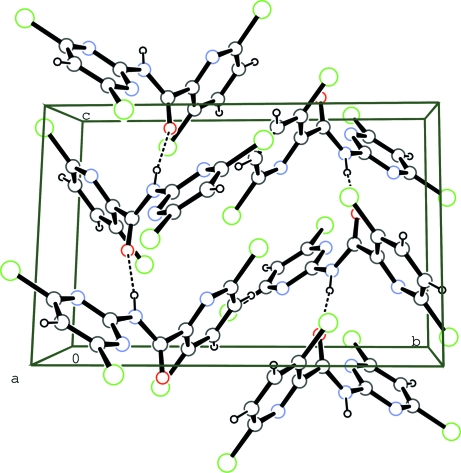
Crystal packing of the title compound, showing the hydrogen bonds as dashed lines.

## supplementary crystallographic information

### Comment

The chemical and pharmacological properties of acid amides have been investigated extensively, owing to their chelating ability with metal ions and to their pltentially beneficial chemical and biological activities (Liu et al., 2005; Śladowska et al., 1999). As part of our studies of the synthesis and characterization of these compounds, we report here the synthesis and crystal structure of 3,6-dichloro-N-(4,6-dichloropyrimidin-2-yl)picolinamide. The C=O bond length is 1.208 (3) Å, indicating that the molecule is in the keto form. In the crystal structure, intermolecular N—H···O hydrogen bonds link the molecules into extended chains (Table 1 and Fig. 2). The dihedral angle between the two rings is 86.60 (10)°, which is close to 90 °. The two rings are nearly perpendicular to one another, which keeps the steric effects between these rings least.

### Experimental

A solution of 3,6-dichloropicolinoyl chloride (10 mmol) in 50 ml ethanol was added to a solution of 4,6-dichloropyrimidin-2-amine (10 mmol) in 10 ml ethanol. The reaction mixture was refluxed for 1 h with stirring. Then the resulting pale yellow precipitate was obtained by filtration, washed several times with ethanol and dried *in vacuo* (yield 90%). Analysis calculated for C_10_H_4_Cl_4_N_4_O: C 35.54, H 1.19, Cl 41.96, N 16.58, O 4.73%; found: C 35.51, H 1.21, Cl 41.90, N 16.58, O 4.79%. A methanol solution of the title compound was slowly evaporated and white crystals were obtained after one weeks.

### Refinement

H atoms were positioned geometrically (C—H = 0.93 and N—H = 0.86 Å) and were refined as riding, with *U*_iso_(H) = 1.2*U*_eq_(C, N).

### Crystal data

**Table:**

C_10_H_4_Cl_4_N_4_O	*F*_000_ = 672
*M**_r_* = 337.97	*D*_x_ = 1.773 Mg m^−^^3^
Monoclinic, *P*2_1_/*c*	Mo *K*α radiation λ = 0.71073 Å
Hall symbol: -P 2ybc	Cell parameters from 5203 reflections
*a* = 10.9313 (13) Å	θ = 2.5–27.9º
*b* = 13.3682 (14) Å	µ = 0.93 mm^−^^1^
*c* = 9.3846 (10) Å	*T* = 293 (2) K
β = 112.576 (1)º	Block, colorless
*V* = 1266.3 (2) Å^3^	0.48 × 0.43 × 0.40 mm
*Z* = 4	

### Data collection

**Table:**

Bruker SMART 1000 diffractometer	2494 independent reflections
Radiation source: fine-focus sealed tube	2132 reflections with *I* > 2σ(*I*)
Monochromator: graphite	*R*_int_ = 0.032
*T* = 293(2) K	θ_max_ = 26.0º
φ and ω scans	θ_min_ = 2.0º
Absorption correction: multi-scan(*SADABS*; Sheldrick, 1996)	*h* = −13→13
*T*_min_ = 0.648, *T*_max_ = 0.690	*k* = −14→16
15165 measured reflections	*l* = −11→11

### Refinement

**Table:**

Refinement on *F*^2^	Secondary atom site location: difference Fourier map
Least-squares matrix: full	Hydrogen site location: inferred from neighbouring sites
*R*[*F*^2^ > 2σ(*F*^2^)] = 0.033	H-atom parameters constrained
*wR*(*F*^2^) = 0.096	*w* = 1/[σ^2^(*F*_o_^2^) + (0.054*P*)^2^ + 0.512*P*] where *P* = (*F*_o_^2^ + 2*F*_c_^2^)/3
*S* = 1.02	(Δ/σ)_max_ < 0.001
2494 reflections	Δρ_max_ = 0.34 e Å^−^^3^
172 parameters	Δρ_min_ = −0.30 e Å^−^^3^
Primary atom site location: structure-invariant direct methods	Extinction correction: none

### Special details

**Table:**

Geometry. All e.s.d.'s (except the e.s.d. in the dihedral angle between two l.s. planes) are estimated using the full covariance matrix. The cell e.s.d.'s are taken into account individually in the estimation of e.s.d.'s in distances, angles and torsion angles; correlations between e.s.d.'s in cell parameters are only used when they are defined by crystal symmetry. An approximate (isotropic) treatment of cell e.s.d.'s is used for estimating e.s.d.'s involving l.s. planes.
Refinement. Refinement of *F*^2^ against ALL reflections. The weighted *R*-factor *wR* and goodness of fit *S* are based on *F*^2^, conventional *R*-factors *R* are based on *F*, with *F* set to zero for negative *F*^2^. The threshold expression of *F*^2^ > σ(*F*^2^) is used only for calculating *R*-factors(gt) *etc*. and is not relevant to the choice of reflections for refinement. *R*-factors based on *F*^2^ are statistically about twice as large as those based on *F*, and *R*- factors based on ALL data will be even larger.

### Fractional atomic coordinates and isotropic or equivalent isotropic displacement parameters (Å^2^)

**Table:**

	*x*	*y*	*z*	*U*_iso_*/*U*_eq_	
Cl1	0.35795 (6)	0.27703 (5)	−0.13818 (7)	0.05603 (19)	
Cl2	0.62475 (7)	0.54097 (5)	0.43365 (8)	0.0654 (2)	
Cl3	1.09988 (5)	0.21157 (5)	−0.02538 (7)	0.05207 (18)	
Cl4	1.02903 (6)	−0.06103 (4)	0.35461 (7)	0.05590 (19)	
N1	0.74081 (16)	0.23149 (13)	0.16663 (19)	0.0373 (4)	
H1	0.7257	0.2261	0.2497	0.045*	
N2	0.62326 (16)	0.40893 (13)	0.23044 (19)	0.0391 (4)	
N3	0.87749 (16)	0.09425 (12)	0.24523 (19)	0.0365 (4)	
N4	0.90814 (16)	0.21391 (12)	0.07244 (19)	0.0345 (4)	
O1	0.65761 (15)	0.30109 (12)	−0.07433 (17)	0.0477 (4)	
C1	0.65957 (19)	0.29263 (15)	0.0547 (2)	0.0343 (4)	
C2	0.56552 (19)	0.35255 (15)	0.1045 (2)	0.0337 (4)	
C3	0.4300 (2)	0.35264 (16)	0.0218 (2)	0.0377 (4)	
C4	0.3507 (2)	0.41321 (17)	0.0706 (3)	0.0454 (5)	
H4	0.2592	0.4137	0.0178	0.054*	
C5	0.4096 (2)	0.47242 (16)	0.1984 (3)	0.0453 (5)	
H5	0.3596	0.5148	0.2335	0.054*	
C6	0.5450 (2)	0.46693 (16)	0.2728 (2)	0.0409 (5)	
C7	0.84622 (18)	0.17692 (14)	0.1588 (2)	0.0326 (4)	
C8	1.01267 (19)	0.16221 (16)	0.0782 (2)	0.0356 (4)	
C9	1.0576 (2)	0.07605 (16)	0.1624 (2)	0.0410 (5)	
H9	1.1317	0.0412	0.1640	0.049*	
C10	0.9829 (2)	0.04607 (15)	0.2440 (2)	0.0372 (4)	

### Atomic displacement parameters (Å^2^)

**Table:**

	*U*^11^	*U*^22^	*U*^33^	*U*^12^	*U*^13^	*U*^23^
Cl1	0.0484 (3)	0.0739 (4)	0.0436 (3)	−0.0043 (3)	0.0151 (3)	−0.0118 (3)
Cl2	0.0771 (4)	0.0715 (4)	0.0639 (4)	−0.0244 (3)	0.0452 (4)	−0.0292 (3)
Cl3	0.0420 (3)	0.0729 (4)	0.0502 (3)	0.0091 (2)	0.0275 (3)	0.0117 (3)
Cl4	0.0705 (4)	0.0384 (3)	0.0589 (4)	0.0150 (3)	0.0248 (3)	0.0111 (2)
N1	0.0406 (9)	0.0437 (10)	0.0356 (9)	0.0093 (7)	0.0234 (8)	0.0071 (7)
N2	0.0414 (9)	0.0450 (10)	0.0380 (9)	−0.0001 (8)	0.0231 (8)	−0.0001 (8)
N3	0.0422 (9)	0.0331 (9)	0.0357 (9)	0.0000 (7)	0.0166 (7)	0.0004 (7)
N4	0.0349 (8)	0.0366 (9)	0.0352 (9)	0.0011 (7)	0.0170 (7)	−0.0012 (7)
O1	0.0500 (9)	0.0659 (10)	0.0353 (8)	0.0160 (7)	0.0253 (7)	0.0077 (7)
C1	0.0358 (10)	0.0388 (11)	0.0343 (10)	0.0023 (8)	0.0200 (8)	0.0010 (8)
C2	0.0375 (10)	0.0354 (10)	0.0331 (10)	0.0065 (8)	0.0189 (8)	0.0065 (8)
C3	0.0408 (10)	0.0422 (11)	0.0321 (10)	0.0047 (9)	0.0162 (9)	0.0052 (8)
C4	0.0371 (11)	0.0534 (13)	0.0467 (12)	0.0141 (9)	0.0173 (9)	0.0106 (10)
C5	0.0550 (13)	0.0412 (12)	0.0508 (13)	0.0137 (10)	0.0327 (11)	0.0063 (10)
C6	0.0515 (12)	0.0377 (11)	0.0434 (11)	−0.0025 (9)	0.0292 (10)	0.0000 (9)
C7	0.0335 (9)	0.0336 (10)	0.0319 (9)	0.0006 (8)	0.0139 (8)	−0.0026 (8)
C8	0.0329 (9)	0.0437 (11)	0.0310 (9)	−0.0007 (8)	0.0132 (8)	−0.0039 (8)
C9	0.0372 (11)	0.0425 (12)	0.0431 (11)	0.0085 (9)	0.0153 (9)	−0.0033 (9)
C10	0.0421 (11)	0.0321 (10)	0.0331 (10)	0.0042 (8)	0.0098 (9)	−0.0023 (8)

### Geometric parameters (Å, °)

**Table:**

Cl1—C3	1.729 (2)	N4—C7	1.335 (2)
Cl2—C6	1.734 (2)	O1—C1	1.208 (2)
Cl3—C8	1.732 (2)	C1—C2	1.512 (3)
Cl4—C10	1.726 (2)	C2—C3	1.383 (3)
N1—C1	1.358 (3)	C3—C4	1.387 (3)
N1—C7	1.389 (2)	C4—C5	1.373 (3)
N1—H1	0.8600	C4—H4	0.9300
N2—C6	1.323 (3)	C5—C6	1.375 (3)
N2—C2	1.338 (3)	C5—H5	0.9300
N3—C10	1.324 (3)	C8—C9	1.376 (3)
N3—C7	1.335 (3)	C9—C10	1.376 (3)
N4—C8	1.319 (3)	C9—H9	0.9300
			
C1—N1—C7	125.77 (15)	C4—C5—C6	117.70 (19)
C1—N1—H1	117.1	C4—C5—H5	121.1
C7—N1—H1	117.1	C6—C5—H5	121.1
C6—N2—C2	117.08 (17)	N2—C6—C5	124.9 (2)
C10—N3—C7	114.73 (16)	N2—C6—Cl2	115.41 (16)
C8—N4—C7	114.49 (17)	C5—C6—Cl2	119.64 (16)
O1—C1—N1	125.91 (17)	N4—C7—N3	127.17 (17)
O1—C1—C2	120.51 (18)	N4—C7—N1	117.50 (17)
N1—C1—C2	113.57 (15)	N3—C7—N1	115.27 (16)
N2—C2—C3	122.36 (17)	N4—C8—C9	125.06 (18)
N2—C2—C1	115.17 (17)	N4—C8—Cl3	115.62 (15)
C3—C2—C1	122.40 (18)	C9—C8—Cl3	119.29 (15)
C2—C3—C4	119.07 (19)	C10—C9—C8	113.97 (18)
C2—C3—Cl1	121.29 (15)	C10—C9—H9	123.0
C4—C3—Cl1	119.62 (17)	C8—C9—H9	123.0
C5—C4—C3	118.8 (2)	N3—C10—C9	124.56 (19)
C5—C4—H4	120.6	N3—C10—Cl4	116.92 (16)
C3—C4—H4	120.6	C9—C10—Cl4	118.52 (16)
			
C7—N1—C1—O1	8.1 (3)	C4—C5—C6—N2	0.1 (3)
C7—N1—C1—C2	−172.25 (18)	C4—C5—C6—Cl2	179.38 (17)
C6—N2—C2—C3	−1.0 (3)	C8—N4—C7—N3	−1.4 (3)
C6—N2—C2—C1	176.22 (17)	C8—N4—C7—N1	175.62 (17)
O1—C1—C2—N2	−124.1 (2)	C10—N3—C7—N4	0.9 (3)
N1—C1—C2—N2	56.2 (2)	C10—N3—C7—N1	−176.17 (17)
O1—C1—C2—C3	53.0 (3)	C1—N1—C7—N4	29.0 (3)
N1—C1—C2—C3	−126.6 (2)	C1—N1—C7—N3	−153.63 (19)
N2—C2—C3—C4	0.0 (3)	C7—N4—C8—C9	0.9 (3)
C1—C2—C3—C4	−176.93 (18)	C7—N4—C8—Cl3	−177.03 (14)
N2—C2—C3—Cl1	−178.64 (15)	N4—C8—C9—C10	−0.1 (3)
C1—C2—C3—Cl1	4.4 (3)	Cl3—C8—C9—C10	177.81 (15)
C2—C3—C4—C5	1.0 (3)	C7—N3—C10—C9	0.1 (3)
Cl1—C3—C4—C5	179.68 (17)	C7—N3—C10—Cl4	179.59 (14)
C3—C4—C5—C6	−1.0 (3)	C8—C9—C10—N3	−0.5 (3)
C2—N2—C6—C5	0.9 (3)	C8—C9—C10—Cl4	−179.95 (15)
C2—N2—C6—Cl2	−178.40 (14)		

### Hydrogen-bond geometry (Å, °)

**Table:**

*D*—H···*A*	*D*—H	H···*A*	*D*···*A*	*D*—H···*A*
N1—H1···O1^i^	0.86	2.09	2.937 (2)	170

Symmetry codes: (i) *x*, −*y*+1/2, *z*+1/2.
